# Expansion of Non-Native Brown Trout in South Europe May Be Inadvertently Driven by Stocking: Molecular and Social Survey in the North Iberian Narcea River

**DOI:** 10.3390/ijms160715546

**Published:** 2015-07-09

**Authors:** Jose L. Horreo, David Abad, Eduardo Dopico, Maud Oberlin, Eva Garcia-Vazquez

**Affiliations:** 1 Department of Ecology and Evolution, University of Lausanne, Lausanne 1015, Switzerland; 2 Department of Functional Biology, University of Oviedo, C/Julian Claveria s/n, Oviedo 33006, Spain; E-Mails: dabad87@gmail.com (D.A.); maud_oberlin@hotmail.fr (M.O.); egv@uniovi.es (E.G.-V.); 3 Department of Education Sciences, University of Oviedo, C/Aniceto Sela s/n, Oviedo 33005, Spain; E-Mail: dopicoeduardo@uniovi.es

**Keywords:** *Salmo trutta*, hatchery, stocking, introgression, management

## Abstract

The biological and anthropogenic (management) factors that may contribute to the expansion of non-native lineages in managed fish have been studied in this work taking brown trout (*Salmo trutta*) as a model species. The changes of users’ opinion about stocking was studied employing social science methodology (surveys). The evolution of hatchery stocks together with the outcome of stocking were analysed with two genetic tools: the LDH-C1* locus (marker of non-native stocks) and six microsatellite loci (for assignment of wild trout to the natural population or putative hatchery stocks). Consulted stakeholders were convinced of the correctness of releasing only native stocks, although in practice the hatcheries managed by them contained important proportions of non-native gene carriers. Our results suggest that allochthonous individuals perform better and grow faster in hatchery conditions than the native ones. We also find a dilution of the impact of this kind of suplementation in wild conditions. The use of only native individuals as hatchery breeders tested for the presence of non-native alleles previously to the artificial crosses must be a priority. Surveys can help steer policy making toward decisions that will be followed by the public, but they should not be used to justify science.

## 1. Introduction

Introduction of non-native genes in wild populations is widely recognized as a major threat for both genetic variability and biodiversity, and fish are not exceptions [1]. Many countries have adopted regulations to restrict or completely ban release of foreign species and non-native stocks in natural ecosystems (e.g., Spain). However, releases of domestic (hatchery-reared) individuals are still common despite that their real contribution to incrementing wild population censuses is doubtful. For example, high numbers of hatchery-reared Pacific salmon have been released in North American river basins, and may be hindering the recovery of depleted populations since they negatively correlate with survival in chinook salmon [2]. Wild and cultured subpopulations should be managed separately for both harvest and reproduction, in order to avoid losses of local adaptations and outbreeding depression, proccesses that are well documented in centrarchids and salmonids [3].

In Spain, as in other countries (e.g., [4]), wild brown trout populations have been supplemented with non-native stocks from northern European countries [5,6]. As a consequence, introgression of foreign genomes into wild gene pools has produced a serious erosion of their ancient lineage diversity [7,8]. In the north of the Iberian Peninsula (Spanish region of Asturias) this practice is currently illegal. Imports of foreign stocks were officially halted in 1992, and releases of non-native specimens have been forbidden since 2007 for preserving authochthonous diversity (Article 52.2, Law 42/2007 of Natural Patrimony and Biodiversity). Despite this, the frequency of non-native specimens within wild populations has increased since 1992 [9]. Moreover, illegal stocking has been recently detected in protected areas and traced by DNA methodology [10].

Surveillance for preventing actions against diversity and natural patrimony, together with increased and effective fines to infractors, are two possible ways for controlling stocking and illegal fisheries. However, other alternatives may yield better results. Water resources and wild areas management have to take into account local stakeholders (e.g., [11,12] and many others). Engagement of local actors is especially important in fishery management, because of the accentuated worldwide decline of wild fish populations, leaving most fisheries still unassessed [13]. This is the case of northern Spanish native salmonids (Atlantic salmon *Salmo salar* and brown trout *S. trutta*): their actual exploitation rate is still unknown despite their wild populations have been declining in the last decades (e.g., [14,15]). It is important to note here that a long-term population size balance between their sympatric populations exist in this region [16]. In Asturias the participation of locals is encouraged and welcomed by the managers responsible for wild freshwater resources (the Regional Government). Local associations of anglers can actively participate in wild salmonid populations management under the “collaborator society” figure (a private fishermen association that release stocked fish in collaboration with the government). For all these reasons, the study of the users’ opinion regarding species’ management is essential for effectively implementing changes in management and improving conservation actions.

In this study we have investigated how anthropogenic management contributes to the expansion of non-native lineages in supportive breeding proccesses. Spanish brown trout and the evolution of non-native alleles in hatchery stocks were analysed on the River Narcea. Two different genetic tools were employed for this purpose: (a) the LDH-C1* locus (an adaptative locus encoding the lactate dehydrogenase protein) as a marker of native/non-native stocks [5,6,9] and (b) microsatellite (neutral) loci for assignment of wild trout to the natural wild population or to the putative hatchery stocks. The changes of users’ opinion about stocking as a solution for salmonids’ population decrease were also studied employing social science methodology (surveys).

## 2. Results

### 2.1. User’s Opinions about Salmonid Current Trends and Management Strategies

**Figure 1:**
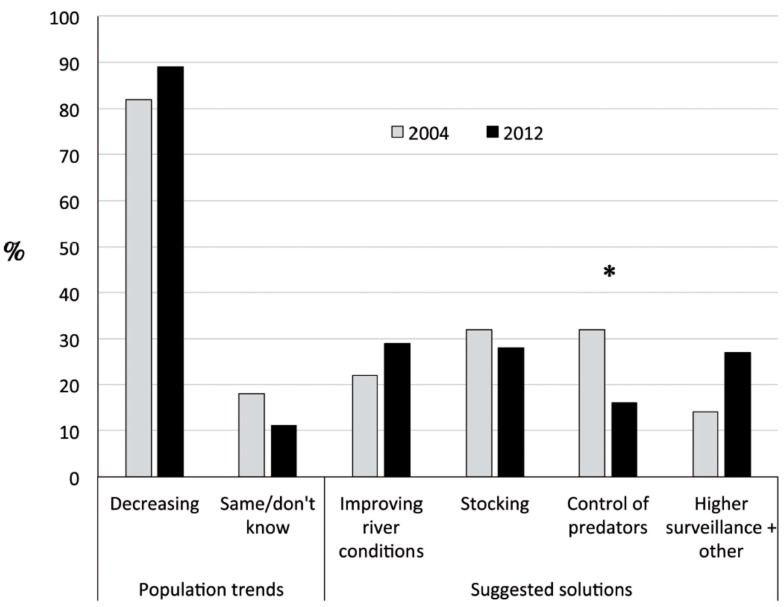
Results of the social survey in 2004 and 2012. Perception of local users of the river about the trend of Salmonid populations and possible solutions for improving their status, presented as percentage (%) over the total number of answers obtained for each question. Significant (*p* < 0.05) differences between 2004 and 2012 answers are marked by an asterisk.

Stakeholder’s perceptions did not change very much during the eight-year period considered (Figure 1). In 2004 and 2012, the majority of them considered that Salmonid populations were in decline in the Narcea River (82% and 89% respectively). More frequently proposed solutions for stopping population declines were stocking (31%) and control (by elimination) of predators (33%) in 2004. However, in 2012 the improvement of river conditions (control of pollution and habitat restoration) was the most frequent proposal (29%), shortly followed by stocking (28%). Control of fish predators was relegated to the last place in 2012 (16%). In this sense the cormorant was the most cited predator species both years. Differences between the 2004 and 2012 surveys were statistically significant for one of the proposed solutions, control of predators (Chi-Square of contingency of 4.25, 1 d.f., *p* < 0.05), but not for the rest (Chi-Square values of 0.31, 0.81 and 2.92 for improving river conditions, stocking and increasing surveillance, respectively; all <3.84 as the threshold for *p* < 0.05 and therefore not significant). The perception of declining populations was not significantly different between 2004 and 2012 (Chi-Square of 1.2, 1 d.f., *p* = 0.2, not significant).

Interviews with the presidents of the five fishermen associations involved in the Narcea–Nalon basin management (included in the data presented in Figure 1) consistently revealed that the official position of the local associations is to use only native individuals as breeders in supportive stocking. The reason given by all of them was better adaptation of natives to local river conditions.

### 2.2. Non-Native Brown Trout Alleles in Hatchery Conditions

All except one hatchery stock used for releases of brown trout juveniles in the High Narcea (hatchery F) contained a proportion of individuals carrying the non-native LDH-C1*90 allele (Table 1). The introduction of native local breeders into hatchery stocks represents a directional selection against non-native alleles, reflected in a decreased frequency of the *90 allele in the hatcheries with temporal replicates (I and N). It must be noted that in the year 1991, Asturian hatchery stocks contained principally non-native trout with the *90 allele almost fixed (*q* approx. 1; [5]). Hatcheries I, E and N have produced juveniles for more than 10 years and have interchanged breeders among them in the past. However, the stock of hatchery F is relatively recent (year 2006), and it was created with local adults from upstream areas. This explains the absence of non-native alleles therein.

Despite the introduction of local native individuals in hatchery stocks, non-native alleles have not been totally removed from the majority of them. The outcome of LDH-C1*90 carriers in hatchery conditions, examined in Hatchery I in 2012, suggests a positive selection in hatchery for this allele (Table 2). First, its frequency increased from 0.27 in the breeders (Table 1) to 0.36 and 0.5 in the two batches analyzed (Table 2). This hypothetical selection would occur only in early stages, because it dropped in later samples down to 0.26 and 0.44 respectively. Second, allele *90 carriers experienced higher growth than *100/100 homozygotes when transferred to the tank, in the two replicates. Although in the batch of Replica 1 *100/100 fry were bigger than *90 carriers, the situation changed once transferred to the tank, where they reached rapidly a similar no significantly different size than *100/100 individuals (Table 2). In Replica 2, *90 carriers were bigger and experienced higher growth than non-carriers in all the analyzed samples.

**Table 1:** Frequency of the allele LDH-C1*90, as *q* (*90), in the broodstocks of four hatcheries that carry out juvenile brown trout releases in the High Narcea in different years. *N*, sample size.

Hatchery	Year	*N*	*q* (*90)
I	1997	50	0.518
I	2005	28	0.143
I	2012	53	0.27
E	2004	28	0.422
N	2005	40	0.6
N	2012	158	0.41
F	2008	61	0

**Table 2:** Frequency, size (furcal length in mm) and growth of carriers of the non-native *90 allele in Hatchery I in 2012 (two replicas).

Replica		*q* (90* Allele Frequency)	Size			Growth		
			Non Carriers	Carriers	Comparison	Batch	Non Carriers	Carriers
**Replica 1**	Batch 1	0.36	19.25	19.14	Bigger NC	Batch 1	2.75	2.06
	Batch 2	0.30	22.0	21.20	Bigger NC	Batch 2	3.80	3.6
	Batch 3	0.27	25.8	24.80	Bigger NC	Tank	8.00	8.62
	Tank	0.26	33.8	33.42	N. S.	Total	14.55	14.28
**Replica 2**	Batch 1	0.50	20.2	20.20	N. S.	Batch 1	0.80	1.55
	Batch 2	0.31	21.0	21.75	Bigger C	Batch 2	2.90	2.95
	Batch 3	0.36	23.9	24.70	Bigger C	Tank	11.40	12.00
	Tank	0.44	35.3	36.70	Bigger C	Total	15.10	16.00

### 2.3. Non-Native Brown Trout Alleles in the Wild

In the River Narcea, non-native alleles were not found in the 1990s and early 2000s, representing the largest drainage of the region free of alien genomes [9]. In 2008, however, some *90 alleles were detected in trouts of the studied area inside a Natural Reserve of the Biosphere (Muniellos; [10]). In the whole area sampled, only those four individuals out of 462 (0.87%) possessed a *90 allele (*q* = 0.05). Two of them, both heterozygotes *90/100, could not be assigned to any of the putative hatcheries (see below) and may indicate natural reproduction of non-native lineages in the wild.

The non-native alleles scarce proportion does not mean however that the hatchery releases were unsuccessful. The loci employed in this study for genetic assignment exhibited different variation in the hatchery stocks (Table 3). The High Narcea population was the most variable, as expected from its condition of large natural population. The five putative stocks were all significantly different (Table 4), and self-assignment tests were sufficiently powerful for robust assignment of individuals to putative populations with the methodology of Rannala and Mountain [17] (quality index: 95.4% and 95.8% of individuals correctly assigned). These Rannala and Mountain assignment tests showed a total of 41 individuals (8.9% of the total river sample) assigned to any of the hatcheries F, I and N (Table 5). No one trout was assigned to E. One LDH-C1*90/90 and one heterozygote *90/100 were assigned to I; the other 39 individuals of hatchery origin were all *100/100 homozygotes. A significant negative correlation was found between the frequency of the *90 allele in hatchery stocks and the relative success of the hatcheries, directly measured as percentage of individuals assigned to that hatchery over the total river sample (*r* = −0.971, 2 d.f., *p* < 0.05).

**Table 3:** Genetic variability at microsatellite loci studied by the number of alleles per locus for each population (Na), allelic richness (AR), and expected and observed heterozygosity (He and Ho, respectively); standard deviation (SD) is shown between brackets.

Marker/Stock	I	N	F	E	High Narcea
SSOSL417	13	11	12	5	26
Ssa197	9	8	15	4	16
SSOSL85	12	6	13	6	15
SSOSL311	14	10	11	4	15
SS4	7	7	13	3	19
BFRO 002	4	4	3	3	4
LDH-C1*	2	2	1	2	2
Na mean	8.71 (4.61)	6.85 (3.18)	9.71 (5.44)	3.85 (1.34)	13.86 (8.35)
AR	5.41	4.24	4.73	2.55	5.72
He	0.76 (0.16)	0.72 (0.14)	0.66 (0.33)	0.57 (0.13)	0.68 (0.33)
Ho	0.70 (0.23)	0.57 (0.18)	0.59 (0.29)	0.82 (0.25)	0.59 (0.31)

**Table 4:** Pairwise *F*_ST_ values (below diagonal) and their statistical significance (above diagonal) between the studied hatcheries and the wild individuals of the High Narcea. *** <0.001.

*F*_ST_/*P*-val	I	N	F	E	High Narcea
**I**	/	***	***	***	***
**N**	0.056	/	***	***	***
**F**	0.153	0.149	/	***	***
**E**	0.142	0.189	0.263	/	***
**High Narcea**	0.152	0.133	0.01	0.246	/

**Table 5:** Contribution of hatchery stocks to the High Narcea wild population, estimated by individual assignment tests to putative origin stocks. *N*, number of individuals of each hatchery origin identified from microsatellite data in the High Narcea. Success, percentage over the total number of samples analyzed (462). Mean *q*, weighted average of *q* (frequency of the allele LDH-C1*90) across years for each hatchery and for the whole hatchery samples analyzed.

Hatchery	*N*	Success	Mean *q*
**F**	27	5.90	0
**I**	12	2.61	0.339
**N**	2	0.44	0.448
**E**	0	0	0.422
**Total**	41	8.95	0.375

**Figure 2:**
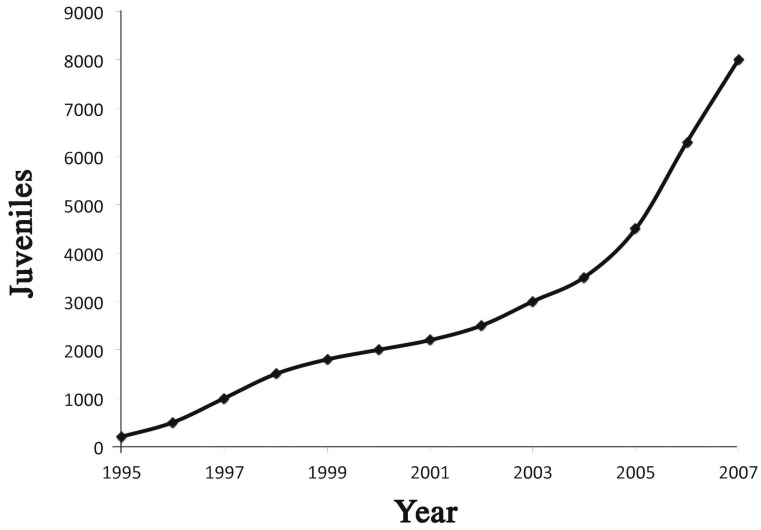
Cumulative stocking in the Narcea River until the year 2008, in thousands of juveniles.

Considering the whole pool of hatchery samples here analyzed (Table 1), the weighted average frequency of the *90 allele was *q* = 0.375 (Table 5). This allele could have been introduced in the High Narcea in any of the years when releases took place, especially in the last years when hundreds of thousands juveniles have been released (Figure 2). In the 41 individuals identified as hatchery descendants (Table 5), this frequency (*90 allele) was very much smaller (*q* = 0.036), being the difference between this and the frequency of the whole pool of hatchery samples highly significant (Chi-Square of 37.95, 1 d.f., *p* < 0.0001). Even considering only the individuals asigned to Hatchery I, which provided the two *90 carriers of this sample of hatchery origin, the q frequency was lower for the 12 individuals found in the High Narcea (0.135) than for those living in the hatchery (0.339 in average).

## 3. Discussion

This study reveals that northern Spain management of brown trout includes repeatedly stocking domestic individuals of mixed gene pool (of non-native origin mixed with local native trout) into wild populations, despite current legislation bans to stock non-native genes. Moreover, regional regulations include the participation of locals on stocking practices, and local stakeholders (from fishermen associations to river users) suggested here alternative or complementary measures that comprise improvement of habitat quality and control of predators. Stakeholders were also convinced of the convenience of releasing only native stocks, although in practice the hatcheries managed by them contain important proportions of non-native gene carriers. Better performance of non-native allele carriers in hatchery conditions would explain their permanence in the Asturian stocks examined in this work.

The validity of single-marker approaches for detecting introgression of non-native lineages has been discussed due to possible different effect of gene drift in the employed markers [8,18]. Selection may also explain discordance between markers of lineage [19]. Since the hatchery stocks here studied contains a mixture of native and non-native lineages, that is, a mixed genetic background, they can be considered a good experimental setting for investigating the selective forces acting on the LDH-C1* locus. Although based in relatively limited sample size, significant and large differences have been found in the outcome of this allele in hatchery, suggesting a positive selection of the *90 allele. LDH-C1* alleles exhibit different kinetic properties that may be of selective value depending on the environmental conditions [20]. Our results suggest that in a protected environment (abundant food, no predators, low current- like in a hatchery) LDH-C1*90 carried may perform better and grow faster thant the others, also indicated by better adaptation of *90 homozygotes to calmed waters like reservoirs [5]. In the river, the presence of the LDH-C1*90 marker was diluted meaning that the impact of this kind of suplementation could disappear in wild conditions, probably because of the reproductive success of the early-generation hatchery fish (half the reproductive success of their wild-origin counterparts when spawning in the wild; [21]) and because of the poorer anti-predatory behaviour and adaptation capacity they have with respect to the wild ones [22]. All these results are only based in a single locus and results must be carefully treated (this phenomena should be ideally studied with thousands of adaptative loci).

The importance of the employement of native wild individuals for creating supportive breeding stocks is widely known despite their problems (e.g., [23]). Our results showed that the hatchery F was the only one without the non-native *90 allele within the stock (Table 5). Moreover, it was the hatchery less genetically differentiated from the wild individuals (Table 4). Both things imply this hatchery performs better than the supportive breeding of the others. Despite the stocking proccess being the same in all the studied hatcheries, the F hacthery differs from the others in its year of creation: it (and its breeders stock) were created more recently than the other hatcheries. In supportive breeding, a fraction of the juveniles is retained every year in the hatchery for renewing the broodstock, and this implies that non-native alleles could be maintained in the hatcheries generation by generation. As mentioned above, the *90 allele suffers dilution in the wild, and this, together with the recent creation of the F hatchery, from which the stock was obtained from the biggest area of the river, could be the reason of the better management done accomplished. A useful initiative for avoiding the maintainence of non-native genes due to supportive breeding could therefore be the complete elimination of previous breeders stocks and the creation of new ones. If they are tested for the presence of non-native alleles previous to the artificial crosses, the stocking proccess would be very much better improved.

This is another interesting case of good-intentioned management measures leading to potentially harmful results for wild populations. In the present case it is clear that local stakeholders have understood the recommendations of scientists concerning stocking measures [5,6,9], and make efforts for stocking with native individuals. This and many other indicators, such as the participation of fishermen associations in the Regional Council of Freshwater Ecosystems, suggest that the pre-requisite of communication patterns for future co-management strategies [24] is already established in the region. However, the pressure of the regional regulations requiring more than 75,000 juveniles released per year makes it difficult to completely replace a stock, since the effort is too great for small local associations. In addition, it is not clear that adding more individuals to a river (always employing native individuals for breedings) is beneficial for its wild population, except if releases do not excede the carrying capacity of the habitat or take place in years of poor environmental conditions [2]. Moreover, other species also inhabit the river, and the delicate equilibrium between predators, prey and competitors may be broken if a species is artificially enhanced for both stocking and/or getting rid of predators (as the results of the survey proposed). An ecosystem approach, agreed by stakeholder community, would be the best option [25], but despite that surveys can help steer policy making toward decisions that will be followed by the public, they should not be used to justify science studies and results.

## 4. Materials and Methods

### 4.1. The Case Study: River and Population Management

**Figure 3:**
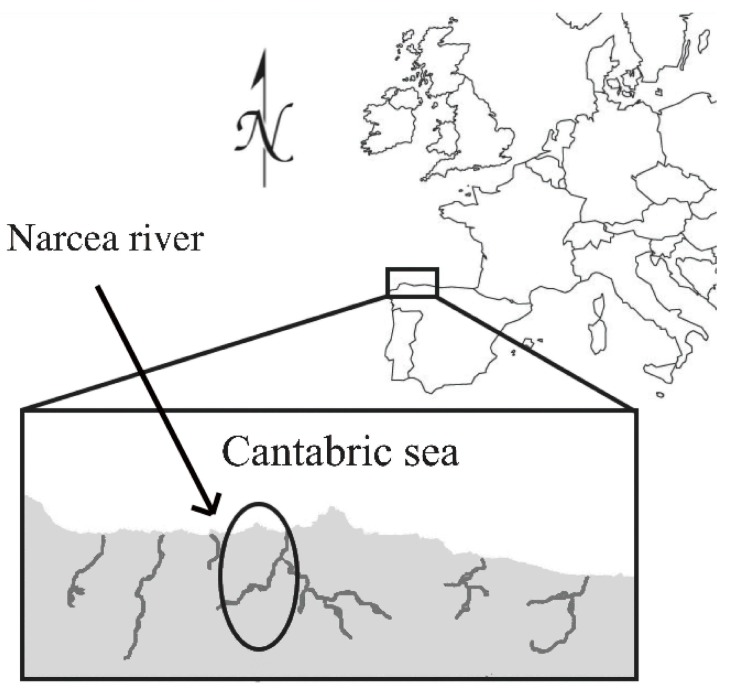
Map of the studied region (north of Spain, Europe) and the river area (High Narcea, in the circle).

The High Narcea is the upper part of the Narcea River, located in the Asturias region of northern Spain (Figure 3). Juveniles from four different hatchery stocks (all the potentially contributing hatcheries, named I, E, N and F as in [10]) have been released in the river since 1993, each hatchery contributing annually with variable quantities. A minimum of 75,000 juveniles per hatchery per year are expected to be stocked in the river (regional management rules). The cumulative total number of juveniles released in the river (years 1995–2007) is presented in Figure 2.

Juveniles released in the river are produced every year from the hatchery broodstock. By artificial spawning, the sperm of at least two males is employed to fertilize the ova of one or a few females and spawnings are kept separately in batches for some weeks. After yolk sac reabsorption, the fry of three, four or five batches are pooled together in larger tanks. During the summer, when the juveniles are six to eight months old, they are released in the river. A fraction of the juveniles is retained every year in the hatchery for renewing the broodstock. New breeders are annually incorporated to the broodstock, obtained by electrofishing from local populations. All Asturian hatcheries were checked for the presence of non-native alleles in 1991 and 2004–2005 [5,10]. Managers make a continuous effort in replacing breeders carrying the LDH-C1*90 allele by native trout adults from local rivers.

Besides stocking, management includes a fishing quota: a maximum of eight trouts per angler per day in 2012. Angling is allowed from March to August. Upstream zones within each tributary are considered high mountain spawning zones and fishing is not allowed there. Surveillance aimed at preventing from illegal fishing is done by staff of the Regional Government (river gards) and by the Spanish national SEPRONA (Servicio de Protección de la Naturaleza = Service of Nature Protection), a police branch in charge of environmental safeguard.

### 4.2. Stakeholders’ Surveys: Methodology

This methodology has been described in a previous work [26]. Briefly, river users (principally anglers) were interviewed on the Narcea River banks while fishing or other activities, employing an Open Response Questionnaire, with open-ended questions where the participants can explain themselves using their own words. In 2012, 79 interviews were done. We present here the results obtained for the questions: “Trends observed in local Salmonid populations during the last years”, and “Solutions suggested for improving/conserving the status of Salmonid populations”. We compared these results with those obtained in 2004 for the same questions (50 interviews in the Narcea River; [26]). In addition, in 2012 the presidents of the five local fishermen associations that collaborate in stocking the Narcea-Nalón River basin (namely Asociación de Pescadores Las Mestas del Narcea, Sociedad de Pescadores Fuentes del Narcea, Sociedad de Pescadores El Banzao, Sociedad de Pescadores Amigos del Nalón, Asociación Allerana de Pescadores El Maravayu) were interviewed in order to know the official opinion of their Association regarding the importance of the hatchery stock origin (native versus non native).

### 4.3. Identification of Individuals from Non-Native Lineages

The native/non-native origin of fish was determined with the LDH-C1* locus, wich encodes the lactate dehydrogenase protein. It was genotyped by PCR methodology following McMeel protocol [27]. This locus has two different alleles: *100 and *90, which have been widely described and employed for distinguishing pure native Asturian and pure non-native brown trout stocks, respectively [5,6,9].

LDH-C1* genotypes were obtained for samples of hatchery breeders taken from the four considered hatcheries in different years. This locus was also analyzed in river samples (see Section 4.4).

### 4.4. Evolution of the Non-Native Allele in Hatchery Conditions

The hatchery I was considered as case study for investigating how individuals carrying native and non-native alleles perform in hatchery conditions. The trait examined was growth, which was measured as furcal length (the distance from the tip of the snout to the shortest fin ray) at age in mm. All breeders employed in the broodstock in 2011 (*n* = 53) were genotyped. Their offspring were sampled at random from two hatchery batches (“replicas”) at the start of the proccess and then each batch was followed and re-sampled two more times, the last one in the moment of tranfer to bigger tanks after yolk sac reabsorption. Trout samples from each tank were measured two weeks later to record growth in the tank. In each sampling, 20 trout were lengthened and the adipose fin clipped for genotyping the LDH-C1* marker.

Allele *90 frequency was calculated from LDH-C1* genotypes for the hatchery samples. Length average and standard deviation were calculated for alevin and juvenile samples. Comparisons between samples or subsamples (*i.e.*, *90 allele carriers versus non carriers) were done by *t*-tests with the software Microsoft Excel.

### 4.5. Assessment of Stocking Success in the High Narcea

High Narcea brown trout population was sampled in 2008. The four hatchery stocks were sampled in the years 2004–2005 by cutting adipose fins and releasing the fish in the same hatchery tanks from where they were taken. Juvenile wild brown trout individuals were sampled by electrofishing along ten different sampled points along the Narcea river covering its major part. A total of 462 wild individuals adipose fin clips were obtained. DNA was extracted following a resine-based protocol [28]. Samples were genotyped for the LDH-C1* locus as explained above in order to determine their native/non-native origin, and for six microsatellite loci (following previous protocols [29]), in order to assign river samples to the different putative stocks: the native High Narcea population or any of the hatcheries that have released juveniles in the river during the previous years. The six microsatellite loci employed were: Ssa197, SSOSL417, SSOSL311 and SSOSL85, SS4 and BFRO002. Their genetic variability (number of alleles, allelic richness, and observed and expected heterozygosity) was estimated with the Arlequin v.3.11 software [30]. The High Narcea samples were previously analyzed for a larger study about population connectivity [29], and the hatchery stocks for tracing illegal stocking [10].

Assignment tests searching for hatchery/wild origin of river individuals were done with GENECLASS2 software [31]. First step was to estimate the genetic differences (*F*_ST_ and their *p*-values) among hatchery stocks (stocks must be genetically different). *F*_ST_ values were estimated with Arlequin v.3.11 [32]. Then, self-assigning of the samples were done for testing the power of the baseline with the two Bayesian criteria for computation implemented in this software: Rannala and Mountain [17] and Baudouin and Lebrun [32]. Results of assignment tests were checked with an exclusion method through the simulation algorithm of Paetkau [33] (10,000 individuals simulated and a type error I (alpha) of 0.01).
